# 

**Published:** 1912-11-09

**Authors:** 


### Answers to Correspondents.

"A Nurse" writes: I am a fully trained hospital nurse. I left my hospital in 1897, and since that date I have been acting as nurse-companion, receiving a salary of £25 a year. I should be very glad to know from you what society you would advise me to join.

You appear to us to come within the class specially catered for by the Nurses' Insurance Society, 15 Buckingham Street, Strand, W.C., and we advise you to apply to the Secretary.

### Doctors' Estates.

Sir William Japp Sinclair, M.D., for some years joint editor of the *Medical Chronicle*, and one of the founders of the *Journal of Obstetrics*, professor of obstetrics and gynæcology at Victoria University, Manchester, who died on August 21, aged sixty-six, left estate of the gross value of £856.

Mr. Leonard Arthur Bidwell, F.R.C.S., of Upper Wimpole Street, W., and of Bexhill, surgeon-major in the Bucks Yeomanry, consulting surgeon to the City Dispensary, dean of the Post-Graduate College at the West London Hospital, has left estate of the gross value of £12,223, of which the net personalty has been sworn at £8,305.

### Buildings Contemplated.

Norwich.—Messrs. Morgan and Buckingham are the architects for additions to the Guardians' premises, for which tenders are being considered. The works include the erection of children's homes and superintendent's residence.

Ribchester.—The Preston Board of Guardians has passed plans and estimates for workhouse alterations to cost £14,070.

Staines.—Tenders are invited for extensive drainage works by the Staines Union and Joint Hospital Committee. Particulars may be obtained from the engineer, Mr. G. W. Manning, London Road, Ashford, Middlesex.

Sunderland.—Additions are contemplated to the Hylton Road, Sunderland, Workhouse Infirmary. Particulars may be obtained from the architects, Messrs. W. and T. R. Milburn, FF.R.I.B.A., of Sunderland.

Tredegar.—Additions are proposed to Tredegar Workhouse for the Bedwellty Guardians. Tenders are being considered on plans prepared by Messrs. James and Morgan, FF.R.I.B.A., of Cardiff.

Ulverston.—Messrs. Settle and Brundrit are architects for additions to the sanatorium at High Carley. Tenders are under consideration.

### Appointments.

The Sanitary Committee of the Leeds Corporation have appointed Dr. H. de C. Woodcock to be chief clerical physician of the Tuberculosis Dispensary.

Mr. V. L. Connolly has been appointed sixth assistant medical officer at Colney Hatch Mental Hospital, in succession to Mr. H. C. Waldo, who has resigned.

Dr. Mabyn Read has been appointed a whole-time medical officer of health to the City of Worcester. He has held the post of hon. physician to the Worcester General Infirmary since 1894.

Professor Robert Gordon McKerron has been appointed professor of midwifery at the University of Aberdeen, in succession to Professor Stephenson, who filled the chair for thirty-seven years till his recent retirement.

The following appointments have been made at the Manchester Royal Infirmary: Mr. E. E. Hughes, F.R.C.S. (Eng.), as surgical registrar; Mr. G. B. Warburton, F.R.C.S. (Eng.), resident medical officer, Barnes Convalescent Home, Cheadle; Mr. James M. Scott, M.A., M.B., Ch.B. (Glasgow), medical officer at the Central Branch; Mr. Samuel Rutherford, M.B., Ch.B. (Glasgow), assistant medical officer, Barnes Convalescent Home, Cheadle; and Mr. Howard Buck, F.R.C.S. (Eng.), has been reappointed resident surgical officer at the Royal Infirmary.

### Obituary.

Dr. Ludovic William Darra Mair, Medical Inspector to the Local Government Board, has died in London, after an operation, at the age of forty-six. After a private education in London and at Epsom College, he studied at St. Bartholomew's Hospital, and graduated M.D. at London University in 1893. After work at Coppice Mental Hospital, Nottingham, and as Medical Officer of Health for Croydon, he was appointed in 1898 Medical Inspector in the Local Government Board Office by Mr. Henry Chaplin. The etiology of infectious disease here occupied his attention, and in 1910 he presented to Parliament a special report on the incidence of disease among dwellers in back-to-back houses. He served on the Commission appointed by the Irish Local Government Board in 1908 to inquire into the causes of the high mortality prevailing in Belfast, and in the investigation of the Lancashire outbreak of arsenic poisoning (in 1900), and was Chairman of the Departmental Committee on the use of intercepting traps in house drains, whose report appeared only this year.

The Chester Town Council decided on Tuesday last to confer the Freedom of the City upon Mr. Albert Wood, of Bodlondeb, Conway, North Wales. Mr. Wood has recently given £12,500 to Chester Infirmary, which is being reconstructed as the local memorial to King Edward.

### BUREAU OF INFORMATION.

### Rules for Correspondents.

1. Every letter, on whatever subject, **must be accompanied by the coupon** to be cut from the back cover (inside page) of The Hospital, current issue, and must contain the name and address of the correspondent with pseudonym for publication if desired.

2. Replies by post cannot be given, save under exceptional circumstances at the Editor's discretion.

3. Letters from Approved Homes in reply to special needs published in the Bureau should state terms and full particulars, and be sent prepaid under cover to the Editor of the Bureau with coupon and name enclosed for identification.

4. Proprietors of Homes which have not yet been entered on the List of Approved Homes, but have spare accommodation likely to suit special needs, are invited to write for an application form for registration. The fee for registration, which includes two announcements of the Home in the Bureau, is 5s.

5. Special needs of every description relating to those who are sick or in difficulties are made known in these columns **free of charge.**

6. All communications to be addressed to The Editor of The Hospital, 28 Southampton Street, Strand, London, W.C., and marked "Bureau of Information."

### Approved Homes.

Note.—*No Home is added to the List without direct medical and other testimony to its good management.*

**Lugano.**—Villa Monteverde, Via Bella Vista, Lugano, Switzerland. Principal: Miss Lorraine Heath. Medical, surgical, obstetric and convalescent cases. Splendid winter climate. English comfortable life abroad. Diet a speciality. Fully trained and certificated nurses.

**Dorset.**—Newland, Sherborne. Principal: Mrs. Marden. Convalescents and those in need of rest and change. Massage as required. Simple life. Moderate terms, specially reduced for nurses. No acute cases.

**Somerset.**—Principals: Dr. and his Wife. One patient received. Neurasthenic case or very slight tubercular, or delicate child. Very healthy locality. Home life. Exceptional experience in tubercular cases.

Miss Green, whose Home at 13 St. George's Road, Worthing, was announced in our last issue, offers good accommodation also for delicate or ailing children.

### Training and Employment.

**Army Nursing.**—A three years' certificate from a general hospital or Poor-Law infirmary of good standing is essential for all candidates for Queen Alexandra's Imperial Military Nursing Service, or any of the allied services, including the Territorial Force. Your partial training will be of no service to you in this direction.—Worried.

**Lady Dispensers.**—A short course of training with a view to passing the examination of the Society of Apothecaries is of very little use, and will not lead on to lucrative employment. As your daughter is so young it would be far better for her to take a three years' course with a view to qualifying for the Minor examination of the Pharmaceutical Society. Write to the Secretary of the Medical School for Women, 8 Hunter Street, London, W.C., and ask her for the names of lady chemists who receive pupils. The Minor examination cannot be taken till the student is twenty-one, so eighteen is exactly the right age to begin the course.—Black Tulip.

**Nursing Pupil Wanted.**—An opening offers for an educated girl, eighteen to twenty, to get preliminary training under exceptionally favourable circumstances. Small salary from commencement, and assistance in taking full training afterwards. Prepaid replies to be directed (under cover of the Editor and marked "Bureau") to Dene.

### Nursing Homes Wanted.

**Brine Baths and Massage.**—Gentleman requires rest treatment for rheumatism, with brine baths, and massage from masseur. Neighbourhood of London preferred. Terms from 10s. to 12s. a day. Replies to Rev. E. M. (130a).

**Open Air Sanatorium for Paying Patient.**—The patient is a lady in need of open-air treatment under medical supervision. She would like a good room, with nursing and medical attendance. Terms from £4 4s. to £5 5s. Replies to Snarky (132a).

**Care of Dipsomaniac.**—The case you mention is most distressing. If the patient has no private means an effort could be made to force her to live where some restraint could be exercised in allowing her to obtain alcohol. We understand she is set against entering a retreat for this class of patient. But we have several private homes on our list where she could be received as an ordinary paying guest at moderate terms. We will forward you replies from any who are prepared to assume charge in this way, and trust you may be able to make a choice and induce the lady to follow your wishes. Replies to J. H. (131a).

**Inexpensive Winter Quarters Abroad.**—We are informed that your best plan would be to go to a convent in any locality in the South of France which you think likely to suit you. The nuns in France, being prohibited from taking pupils, are now offering charming accommodation at very low terms to visitors, with the best of attention in case of illness. Direct your letter to the Sister Superior of the principal convent, and send it under cover to the British Consul of the town you select, with a request that he will forward it to the right quarter.—Emm Dee (124a).

### Nursing in the Empire and Abroad.

**Nursing Institution at Pau.**—There is none supplying British nurses, only one where French nurses are employed. We are informed there is a large demand for nurses during the season (November to May), and a nurse who went out would probably get plenty of employment in a short time. Application should be made to the doctors, of whom there are no fewer than forty-six, and to the clergy, of whom there are five.—F. N.

**More Red Cross Nurses Wanted.**—A high authority writes to us from Sofia that Red Cross nurses are urgently required at the present moment. Needless to say no nurses can go to the seat of war, however, unless sent out under the protection of the Red Cross authorities.

**Urgent Need for Trained Nurse at Suez.**—Further advices from Suez enable us to add to the information given in the Bureau of October 19 relating to the demand for nurses in this place. We understand that since our announcement of this want appeared there have been numerous inquiries on the spot about the probability of nurses coming out. Four confinement cases coming off in the first three months of the year are particularly anxious to secure the services of a trained nurse, as at present only an utterly untrained person is available. The delay (often eighteen hours) in getting nurses from Cairo makes this expedient very hazardous, to say nothing of the expense. Both French and English doctors are ready to give immediate employment. A good and inexpensive home is offered to the nurse between cases by a lady in Suez, and another lady has kindly offered to put her up as a guest for a week after arrival. Needful qualifications: Thorough training, midwifery certificate, cheerful and sympathetic disposition; knowledge of French an advantage. Prepaid letters, sent in separate envelope, under cover to the Editor of the Bureau, addressed to Mrs. N., will be forwarded.

### Needs and Helpers.

**Convalescent Home for Nurse.**—Write to the Matron of the Filstone Home, 26 Bloomsbury Place, Brighton, where your friend would be very comfortable. See also reply to Mrs. T. Tolroy in the Bureau of November 2.—Snarky.

Miss R.—We have sent your address to Anxious, and hope she will communicate with you direct. Very sorry to note contents of your letter, and will do our best to help.

### Letter-Box.

Mrs. A.—We are pleased to learn that the Bureau has attracted patients to your Home, and that, as you say, it has proved "the best advertisement" you have ever had. Some credit must rest with yourself for your promptness in replying to announcements and your strict attention to our very simple rules for regulating correspondence.

Mrs. N.—We thank you for your kind letter. We have noted the name of the mental hospital to which you draw attention, and think there seems to have been grave cause for your complaint.

**A leaflet containing particulars of the Bureau will be forwarded free on application.**

### INSTITUTIONAL HOUSEKEEPING.

NOTICE TO OUR READERS.

The Editor welcomes for Institutional Housekeeping any contribution from those engaged in the work of the commissariat in institutions. All accepted contributions are paid for. Queries answered on any subject relating to the supply, cost, storage, distribution, or preparation of food.

### The Editor's Letter-Box.

### Powers of Insurance Committees.

*To the Editor of* THE HOSPITAL.

### Gifts and Bequests.

MR. AUSTEN CHAMBERLAIN has received £250 from the Union-Castle Steamship Company towards the £100,000 he is raising for the London School of Tropical Medicine.

MR. EDWARD WILKINSON JEFFERYS, late of the Cashiers' Department, Chatham Dockyard, has left the ultimate residue of his estate to accumulate during one life, and then to the trustees of his will for distribution among such hospitals in the Administrative County of London and in such proportions as they shall determine.

MRS. JAMES TAYLOR, a local resident, and her family are paying the cost of the new theatre which forms part of the extension of Chester Royal Infirmary. The theatre will be dedicated to the late Mr. James Taylor, F.R.C.S., well known formerly as senior house surgeon. Mr. Charles Jones, of Rossett, has bequeathed £1,000 to the institution, and the amount now received towards the £40,000 required for the renovation scheme is some £29,000.

MISS LOUISA TWINING, of Kensington, one of the first women to be elected on a Board of Guardians, who died on September 25, aged ninety-one, left estate of the gross value of £18,758. She bequeathed £1,000 to King's College Hospital for the "Twining" Ward; £100 to St. John's Hospital, Twickenham, "which was founded by my sister Elizabeth"; and £500 each to the Metropolitan and National Association for Providing Trained Nurses for the Sick Poor, the Metropolitan Provident Medical Association, the Nursing Sisters of St. John the Divine, and the Kensington District Nursing Association.

